# A Community Grows around the Geysering World of Enceladus

**DOI:** 10.1089/ast.2017.1711

**Published:** 2017-09-01

**Authors:** Carolyn C. Porco

**Affiliations:** University of California, Berkeley, California.; Space Science Institute, Boulder, Colorado.

## Abstract

The discovery by NASA's Cassini mission at Saturn in 2005 of a large plume of material erupting from the south polar terrain of Enceladus, sourced within a subsurface ocean of salty liquid water laced with organic compounds, has brought together scientists from a diverse range of disciplines over the last decade to evaluate this small moon's potential for extraterrestrial life. The collection of papers published today in *Astrobiology,* as the mission draws to a close, is the outcome of our most recent meeting at UC Berkeley in June 2016. Key Words: Enceladus—Enceladus Focus Group—Ocean world—Search for biosignatures. Astrobiology 17, 815–819.

The Cassini mission's discovery, in the opening months of the year 2005, of a plume of icy particles (Porco *et al.,* 2006) and vapor (Dougherty *et al.,* 2006) erupting from the south polar region of the small saturnian moon Enceladus, along with follow-up observations collected later that year (*e.g.,* Hansen *et al.,* 2006; Spencer *et al.,* 2006), led quickly to the suggestion that the phenomenon had its source in reservoirs of liquid water (Porco *et al.,* 2006) laced with organic compounds (Waite *et al.,* 2006). How deep the source region might lie below the surface was not then clear, but it was immediately obvious that, should we be right, we had the tangible expression of a body of water beyond Earth—perhaps one that could support life—within easy reach. The world took notice: the suggestion of an extraterrestrial habitable zone within Enceladus, inferred from Cassini's initial findings and published in *Science* in early 2006, reached the front page of the *New York Times* on March 10, 2006. By that time, it had become patently clear to me that a broad range of scientific disciplines would be required to address the question of whether or not biological activity existed within Enceladus.

So was born the idea to form an informal group of scientists from diverse fields of study who would meet from time to time to discuss the latest results and theories on Enceladus and, as a matter of course, set new research directions aimed at advancing our understanding of the moon's habitability and biological potential. Getting astrobiologists interested was the first step. I contacted Chris McKay at NASA Ames Research Center, who knew the astrobiology community better than I did, and requested his help in putting it together. He agreed. We sent out the word: Our first meeting would occur in the fall of 2006, on the campus of Caltech in Pasadena, California.

The vast majority of the attendees at that first meeting were planetary scientists of various stripes—geologists, geophysicists, spectroscopists, and dynamicists—as well as mission designers, all either offering results or ideas, or both, about how Enceladus might have become so geologically active today, or describing future missions and payloads that could expand on Cassini's discoveries. But I was especially pleased that we managed to collect a handful of attendees who began the work of wondering what kind of life, if any, might be present within Enceladus and how we might go about detecting it.

During the subsequent decade at Saturn, Cassini's remote sensing instruments extensively observed the plume and mapped the moon's surface. The plume was found to vary in mass on a diurnal timescale (Hedman *et al.,* 2013); the phase of the variation was significantly delayed from what would be expected from widening and narrowing vents in response to varying tensional stresses in an elastic ice shell, but no model was conclusive (Nimmo *et al.,* 2014). The spacecraft was flown through the plume about a dozen times, allowing the *in situ* instruments to capture material and determine the composition of both ice particles and vapor (Postberg *et al.,* 2009; Waite *et al.,* 2009). In time, we learned that the source of Enceladus' 100+ geysers that create the plume (Porco *et al.,* 2014), as well as ∼5 GW of thermal radiation (Spencer *et al.,* 2013), all of which emerge from prominent fractures crossing the south polar terrain (SPT), is a salty, variable-thickness, global ocean some kilometers below the surface (Thomas *et al.,* 2015) that reaches its greatest thickness under the SPT. Through these findings, together with more recent Cassini evidence of complex organic molecules (Postberg *et al.,* 2017) and even seafloor hydrothermal activity driven by serpentinization (Hsu *et al.,* 2015; Waite *et al.,* 2017), the story of Enceladus has grown all the richer and has affirmed the moon's status as a prime candidate for astrobiological study and the search for life. No other ocean world in the Solar System beyond Earth has been studied in such depth and detail for so long, nor is any so well understood as Enceladus.

**FIG. 1.**
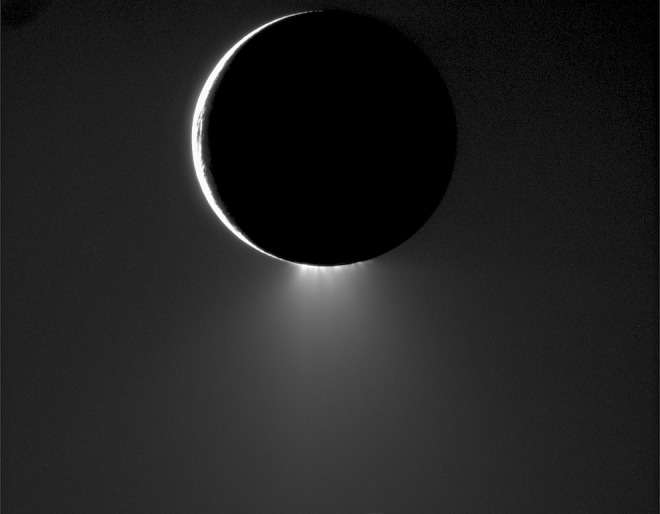
An image of Enceladus and its southern geysers and the faint plume they form, taken by the Cassini high resolution camera on November 1, 2009 through a spectral filter centered at a wavelength of 752 nanometers from a distance of ∼209,000 kilometers. The phase angle is 160^°^. The image scale is ∼1 km/pixel.

Our group continued to meet over the years at roughly 2-year intervals, its membership and discipline representation changing with time. The number of people advising me grew also. By the time of our most recent gathering, in June 2016 on the campus of UC Berkeley, where I had become a visiting scholar in the Astronomy Department, our community had grown substantially. This was, by far, our most wide-ranging, cross-disciplinary meeting to date, and it was thrilling. The usual planetary and astrobiological sciences were well represented, but this time I had managed to lure to the subject organic chemists, microbiologists, microscopists, oceanographers, experts in microfluidics, those studying problems in the origin of life, and even one genomist from the UC Berkeley research lab responsible for the 2016 announcement of a new, far richer tree of life (Hug *et al.,* 2016). Finally, our small, informal focus group had grown to become what I had originally hoped it would be: a place where the usual walls that grow and harden around different scientific disciplines had come crumbling down in the face of an irresistible and real opportunity to test life's distribution in the Cosmos.

The papers gathered together in this special collection devoted to Enceladus, with one exception, resulted from our Berkeley meeting. These works offer a glimpse into the state of a rapidly changing subject at one moment in time … as the Cassini mission ends and we are contemplating the next steps in the exploration of this fantastic little moon. The opening two papers (Barge and White, 2017; Deamer and Damer, 2017) present the examination of two distinct issues, both of extreme importance to the origin of biochemisty. Barge and White focus on the chemistry in hydrothermal vent systems, like those we now believe may be present on the seafloor of Enceladus. These authors ask whether the ability of hydrothermal chimneys to maintain compositional and energy gradients might have played an important role in driving the origin of life and its metabolic characteristics. Deamer and Damer are not pursuing the origin of metabolism, but rather the origin of life's infrastructure and how the basic biomolecules, such as amino acids and other building blocks, could have been concentrated sufficiently to initiate the formation of more complex molecules. The Barge and White study says life requires biologically usable gradients and therefore was more likely to originate where there were plenty of them … oceanic vents. The Deamer and Damer study says that life began when and where the proper building materials could be first assembled, such as hydrothermal springs on the surface that underwent natural cycles of hydration and dehydration. You can read and decide, if you wish, for yourself, or merely be amazed at how far we still are from having a theory for how life began.

The third paper (Benner, 2017) offers a somewhat unusual universal biosignature in the form of testable molecular characteristics that, the author argues, any information-bearing biopolymer must have. An initial proposal along these lines was first made by Schrödinger 70 years ago (Schrödinger, 1943): the various building blocks of a genetic polymer underlying a successful biochemistry must all be similar in size so that they form a regular “crystalline” structure that remains unchanged as the system evolves and the building blocks themselves are rearranged. The second feature, proposed by Benner over a decade ago (Benner and Hutter, 2002), expands on Schrödinger to note that in order to support Darwinian evolution, the physical/chemical behavior of a genetic biopolymer must also remain unchanged as the system evolves. This is achievable in water only if the biopolymer has a backbone with a repeating charge; that is, the biopolymer must be a “polyelectrolyte.” The genetic biopolymers of terran life, that is, the nucleic acids composed of nucleotide building blocks, have such a feature: a backbone of repeating components—the phosphate group—each with an identical charge whose mutual repulsion stretches the molecules; discourages folding; allows templating; and makes it possible for their genetic information, as well as random mutations, to be reliably transcribed from generation to generation. It also allows the overall physical/chemical behavior to persist against structural changes, such as the rearrangement of the building blocks. Benner proposes this as a requirement for any genetic polymer, regardless of the chemical constituents. The advantage of polyelectrolytes is that they are easily concentrated from dilute aqueous solution, like the Enceladus plume. This permits, in practice, the recovery of any universal Darwinian biopolymer using a device no more complicated than a polycharged surface. Maybe some mission back to Enceladus will someday carry such a capability.

**FIG. 2.**
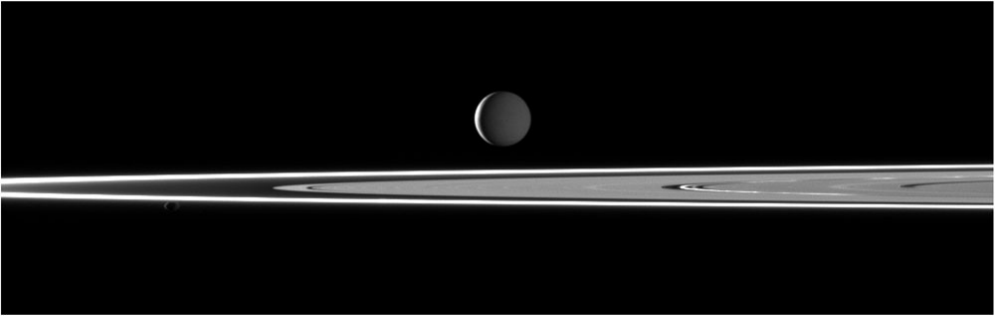
Two light sources illuminate Enceladus in this image taken in visible light with the Cassini high resolution camera on February 11, 2010 from a distance of ∼1.5 million kilometers and a phase angle of 142^°^. Most of the moon is dimly lit by sunlight reflected off Saturn. However, a thin crescent of the moon is directly lit by sunlight. Saturn's bright rings are in the foreground. Image scale is ∼9 km/pixel.

The fourth paper (Judge, 2017) is the one contribution that did not arise from our Berkeley meeting. It dials back the ambition and expense of future explorations of Enceladus and suggests that, to some degree, the chemical makeup of the plume can be investigated, and the search for biomolecules can be conducted, by infrared spectroscopic and spectropolarimetric methods, that is, observing a plume erupting from a body as it transits a bright planetary disk like Saturn, from the largest ground-based telescopes and even eventually the James Webb telescope. Jupiter's 3° obliquity ensures that Europa transits of Jupiter occur every europan day. Enceladus transits of Saturn, whose obliquity is a healthy 27°, are seasonal and next begin in 2022. It is not too early to start making plans.

The four following papers in this collection address, in one form or another, the anticipated bioloads at Enceladus, and the detectable biosignatures that might be present in its plume. Steel *et al.* (2017) construct a thermal model that assumes 10% of the geothermal heat emerging from the moon's core drives hot (90°C) hydrothermal fluid flow, which results, through water/rock interactions, in the production of H_2_. In their work, 100% of the molecular hydrogen thus produced is subsequently consumed by methanogens to produce biomass. They thus estimate, at the vents, 90 μ*M* of biologically produced amino acids, and microbial concentrations as high as 10^9^ cells/mL; 10% of the latter rise in the thermal plumes that originate at the vents and eventually reach the base of the ice shell. If these authors are correct, and Enceladus approaches this high-efficiency scenario, especially if the process of bubble-scrubbing (see below) is at work, then the search for biosignatures, even microbes, in the samples collected from Enceladus' plume could be easily accomplished.

In the Porco *et al.* (2017) study, my coauthors and I photometrically reduce Cassini images to particle number densities at various altitudes in the plume and use the results to estimate the volume of a sample that future Enceladus missions would collect, ranging from single fly-through missions to landers. Like Steel *et al.* (2017), we estimate the bioload at the Enceladus seafloor vents, but unlike Steel *et al.,* we merely scale the bioload at Lost City by the ratio of the geothermal energy flux through the moon's seafloor to that of Earth … a ratio that turns out to be 1. Our results, ∼10^5^ cells/mL, are significantly less optimistic than those of Steel *et al.* However, we introduce to the study of Enceladus, and describe in some detail, the process of bubble-scrubbing, well known to marine microbiologists, whereby microbes and organic matter in natural bodies of water may be found in significantly enhanced concentrations in the spray produced at the surface by breaking bubbles. This outcome follows from the tendency of bubbles, rising in a column of water, to collect available organic material along the way. It is a process that could very well be at work on Enceladus and could make the job of detecting biosignatures within the plume up to a 1000-fold easier.

Mathies *et al.* (2017) describe what can be done with very small sample sizes and microfluidics in determining the concentrations of organic materials in the plume and in determining whether or not particular molecular species show enantiomeric excess, an arguably strong indicator of life. Mathies, the lead author, is one of the earliest developers of microfabricated “lab-on-a-chip” technology and its application to space instruments. He and his coauthors make the case for the scientific feasibility of the Enceladus Organic Analyzer (EOA), a lab on a chip that uses a microfluidic capillary electrophoresis system to sensitively detect a wide range of relevant organic molecules, including amines, amino acids, carboxylic acids, aldehydes, ketones, and thiols with part-per-million sensitivity. The engineering design for both the chip and the capture plate to collect plume particles—not an easy task—are both presented. Now all we need is a spacecraft. And a big rocket. And a bit of funding.

Bedrossian *et al.* (2017) expand on their previous work (Wallace *et al.,* 2015; Lindensmith *et al.,* 2016) in designing and field testing an extraordinary microscopic system being developed at JPL and Caltech: the digital holographic microscope (DHM). True to its name, the DHM is a microscope capable of taking holographic images of a (comparatively) wide field of view, which is necessary for exploratory “survey” work, that capture phase as well as spatial information. The quantitative phase information can be used to distinguish unresolved organisms from bits of inorganic matter; one is made largely of water, the other is not, and so their different indices of refraction make them readily distinguishable. Also, the inclusion of video framing means that any motility would be a discriminating feature as well, made more powerful by the phase capability that allows detection of motion in the third, “out of plane” dimension. Obviously, for a microscope to be fruitful at imaging intact organisms, either plume fly-through speeds must be below ∼2 km/s or the DHM would need to be placed on a lander. A video clip of alien organisms in motion could well be the only unambiguous, 100% confidence-level detection we gather of enceladan life, should it be there at all.

The final two papers in our collection make significant advances in the continued analysis of Cassini data and expand our basic knowledge of the geophysical mechanisms underlying the geysers. Teolis *et al.* (2017) attempt what, to date, has not been done: determine the three-dimensional structure of the vapor component of the plume by comparing, in detail, the vapor measurements by the Cassini Ion and Neutral Mass Spectrometer and the Ultraviolet Imaging Spectrometer with the full, three-dimensional map of the 100+ geysers seen in ISS images of the particle component, with the intent to look for signatures of the latter in the former. Though obvious stochasticity in the vapor emissions makes the job tough, these authors nonetheless are able to say that the structure of the vapor component seems to be best fit with the many-geyser model with some contributions from continuous, inter-geyser emissions along the fractures (Porco *et al.,* 2014). That both types of emissions—discrete and continuous eruptions—are seen in high-resolution Cassini images of the near-surface altitudes of the geysers is corroboration of this conclusion.

Finally, in all previous attempts to explain the diurnal variation of the plume by modeling the cyclical behavior of tidal stresses across the SPT (*e.g.,* Hurford *et al.,* 2013; Nimmo *et al.,* 2014; Běhounková *et al.,* 2015), none were implemented to account for the effect of the fractures themselves on the tidal stress field. The ice shell was treated as a solid, unbroken mass, and the stresses were merely computed at surface locations along the main, geyser-producing fractures and assumed to be the same below the surface. Běhounková *et al.* (2017) take a big step forward in their updated work presented here in devising a numerical method for decoupling one side of a fracture from the other in computing the time-variable stresses. The results are remarkable. Stresses and strains become significantly larger in the vicinity of the fractures, and their model—which still retains some rather simplifying assumptions, such as constant fault-geometry with depth, neglect of stress, friction, and liquid water within the fracture—predicts a spatial distribution of stresses significantly different than that of the nonfracture model. At the moment, the pattern of activity these authors predict does not match the Cassini observations (Porco *et al.,* 2014) in satisfactory detail. But the work is still in its infancy, and I'm sure that, with such powerful new capabilities, it won't be long before this research group will elucidate those factors that are most important in determining the observed distribution of activity across the surface and the precise shape and phase of the plume's diurnal variation in mass.

So, now, I leave you to it. As Cassini's extraordinary 13 years of exploration conclude, enjoy this up-to-the-minute, far-reaching, wide-ranging look at that little moon at Saturn with the big possibilities.
